# Use of Teriparatide in preventing delayed bone healing and nonunion: a multicentric study on a series of 20 patients

**DOI:** 10.1186/s12891-023-06278-0

**Published:** 2023-03-11

**Authors:** Gabriele Gariffo, Vanna Bottai, Federico Falcinelli, Federico Di Sacco, Roberta Cifali, Elisa Troiano, Rodolfo Capanna, Nicola Mondanelli, Stefano Giannotti

**Affiliations:** 1 Second Orthopedic and Traumatology Clinic, University of Pisa, Pisa, Italy; 2 Department of Medicine, Surgery and Neurosciences, University of Siena, Siena, Italy; 3 Section of Orthopedics, Azienda Ospedaliero-Universitaria Senese, Viale Mario Bracci 16, 53100 Siena, Italy

**Keywords:** Nonunion, Anabolic therapy, Delayed union, Fracture healing, Teriparatide, Bone metabolism

## Abstract

**Background:**

Delayed bone healing and nonunions represent a great challenge for the orthopedic surgeon. In addition to traditional surgical approaches, increasing attention is being given to the use of systemic anabolic therapy with Teriparatide, whose efficacy in preventing osteoporotic fractures is widely validated and whose application as a promoter of bone healing has been described but it is still debated. The aim of the study was to evaluate bone healing in a series of patients with delayed unions or nonunions treated with Teriparatide in conjunction with eventual appropriate surgical procedure.

**Methods:**

Twenty patients with an unconsolidated fracture that were treated at our Institutions from 2011 to 2020 with Teriparatide were retrospectively included into the study. The pharmacological anabolic support was used off-label with a planned duration of 6 months; radiographic healing was evaluated at 1-, 3- and 6-months follow-up outpatient visits over plain radiographs. Also, eventual side-effects were registered.

**Results:**

Radiographic signs indicative of favorable evolution of the bone callus were observed as early as at 1 month of therapy in 15% of cases; at 3 months, healing progression was appreciated in 80% of cases and complete healing in 10%; at 6 months, 85% of delayed and nonunions had healed. In all patients, the anabolic therapy was well tolerated.

**Conclusions:**

In accordance to Literature, this study suggests that Teriparatide plays a potentially important role in the treatment of some forms of delayed unions or nou-nions, even in the presence of failure of hardware. The results suggest a greater effect of the drug when associated with a condition in which the bone is in an active phase of callogenesis, or with a “revitalizing” treatment which represents a local (mechanical and/or biological) stimulus to the healing process. Despite the small sample size and the variety of cases, the efficacy of Teriparatide in treating delayed unions or nonunions emerged, highlighting how this anabolic therapy can represent a useful pharmacological support in the treatment of such a pathology. Although the results obtained are encouraging, further studies, particularly prospective and randomized, are needed to confirm the efficacy of the drug, and define a specific treatment algorithm.

## Introduction

Delayed bone healing and nonunion represent today some of the greatest challenges for orthopedic surgeons [1, 2]. Nonunion of bone is the body's inability to heal a fracture. The most agreed-upon standard definition of nonunion made by the Food and Drug Administration (FDA) is a fracture that persists for a minimum of nine months without signs of healing for three months [3, 4]. In addressing these issues, in addition to traditional surgical approaches, increasing attention is being paid to the possibility of using systemic anabolic therapy with Teriparatide. Teriparatide is the synthetic recombinant 1–34 N-terminal peptide of human parathyroid hormone, approved by the FDA for its effect in the treatment of osteoporosis as in increasing the bone mass and in preventing osteoporotic bone fractures [5–7]. While the use and the efficacy of Teriparatide in this field is widely validated, its use as a promoter of fracture healing and in the prevention of complications has been proposed and it is still debated [1, 8–10].

Numerous studies conducted both in vitro and in vivo in animal models have shown a positive effect of this drug with improvement in both the volume and mineralization of bone callus, as well as an increase in the mechanical strength of the bone [11–14]. Despite the lack of randomized controlled trials in humans, there is a growing presence in the Literature of case reports and case series that highlight a potentially important role in the treatment of some forms of delayed union and nonunions, also in the presence of failed hardware, as well as complex fractures [15, 16] particularly where bone metabolism is compromised [17–20].

The aim of this paper is to present the results of a series of patients with delayed unions and nonunions treated with Teriparatide associated with eventual appropriate surgical procedure in two Hospitals. At both our Institutions, no Institutional Review Board nor Ethical Committee Approval is necessary for retrospective studies. Patients gave their consent to data collection and anonymous use of them for scientific and teaching purposes.

## Materials and methods

In 2011 at the University Hospital of Pisa, the administration of Teriparatide to patients with a variety of pathologies (difficult-to-treat fractures, fractures in patients with suspected or diagnosed impaired bone biology, aseptic or sterilized delayed bone healing or nonunions) different from registered indications for the drug was started [11, 14]. Same approach was introduced at the University Hospital of Siena in 2016 [15, 16, 21]. No Ethical Committee approval is necessary for such a sporadic off-label use of already registered drugs at both Institutions. Such a prescription is made under direct medical responsibility previous patient’s information and consent. From the combined and shared database of patients undergoing off-label administration of Teriparatide, a retrospective extrapolation of patients treated between 2011 and December 2020 for unconsolidated fractures with delayed unions or nonunions was conducted. Inclusion criteria for the present study were diagnosis of delayed unions or nonunions after surgical treatment for fractures of long bones done at our Institutions, patients adult of age and sound of mind, and completion of a 6-months follow-up period. Exclusion criteria were delayed unions or nonunions of short bones, infection as a cause (or a contributing cause) of the pathology, patients with history of active or previous oncologic disease, primary hyperparathyroidism, and other contraindications as on the drug data sheet.

Patients’ past and present medical history was assessed, with particular attention to the presence of osteoporosis, diabetes and history of previous fracture(s), which all represent important risk factors for fractures and nonunions [22–24]. Smoking and alcohol abuse, as well as current medical therapies, were also assessed. In particular, drugs with potential action on bone metabolism such as bisphosphonates (BPs) and glucocorticoids (GCs) were considered in order to evaluate any risk factors for delays in bone healing [25–27].

All patients were subjected to a full panel of blood tests and were studied for phosphocalcic metabolism (Table 1). Also, mineralometric evaluation was conducted in all patients. Before starting treatment, written informed consent was acquired from all patients, specifically with regards to the off-label use of the drug, and data were prospectively collected in a database. Teriparatide dosing schedule consisted in 20 μg/day subcutaneously [28], until bone healing would have occurred, and for a maximum of 24 months [29, 30] In addition, all patients received supplementary vitamin D with variable dosage with respect to blood levels. Calcium supplement was prescribed when dietary introduction was considered insufficient (less than 1.000 mg per day).

**Table 1:** Suggested “short” phosphocalcic metabolic panel, including only blood testing without any precise preparation nor a 24-h urine collection

B-ALP, U/L (range 55 – 142)
Ca, mg/dL (range 8.9 – 10.1)
P, mg/dL (range 2.5 – 4.5)
PTH, pg/mL (range 15 – 65)
CTX, ng/L (range 100 – 700, over 50 years)
P1NP, μg/L (range 15 – 75, over 50 years)
25(OH)D, ng/mL (range 30 – 100)
Creatinine, mg/dL (range 0.6 – 1.1)

*B-ALP* Bone-specific isoenzyme Alkaline phosphatase, *Ca* Calcium, *P* Phosphorus, *PTH* Parathyroid hormone, *CTX* C‐telopeptide of type I collagen, *P1NP* Aminoterminal pro-peptide of type I procollagen, *25(OH)D* Cholecalciferol (vitamin D3)

All patients were assessed at 1, 3, and 6 months after the start of the therapy with Teriparatide. Plain radiographs in the antero-posterior and lateral views were obtained at each outpatient visit and were independently evaluated in each center. New bone appearance at cortical site were considered, and fracture healing was defined in accordance with the modified radiographic union scale in tibial fractures (mRUST) score [31–33].

Statistical analysis was performed with the Mann–Whitney U test and the Kruskal–Wallis ANOVA, used for continuous parameters, and the Fisher’s exact test with the Freeman-Halton extension, used for categorical parameters. The significance level was set at *p* < 0.05; *p* was reported as not significant (n.s.) in text and with exact values in tables. All data were elaborated with XlsStat 2020 software (Addinsoft, New York City, NY) for MS Excel (Microsoft, Seattle, WA, USA).

## Results

Twenty patients met the inclusion criteria: there were 12 males (60%) and 8 females (40%) with a mean age ± standard deviation at intervention of 55.3 ± 20.9 years (range 18–82 years). Pathology was localized to the lower limb in 16 cases (80%) including 13 to the femur, and 3 to the tibia, and to the upper limb in 4 cases (20%) including 1 to the humerus and 3 to the forearm. Patients demographics and clinical data are shown in Table 2. Differences were identified among subgroups, with regards to demographics and comorbidities. There was a statistically significant difference between the mean ages of males (46.8 ± 19.3 years) and females (68.0 ± 17.0 years) (*p* = 0.025), and in the prevalence of osteoporosis between genders with a higher frequency in females (*p* = 0.0007). When comparing the mean ages of patients with and without comorbidities (smoke, alcohol abuse, diabetes, previous fractures, osteoporosis), the difference became statistically significant only when considering osteoporosis (73.5 ± 11.1 vs 47.4 ± 19.2, *p* = 0.0065) (Table 3). Delayed union was present in 95% of cases (19 out of 20 patients), while in one case a nonunion of an atypical femoral fracture was present. The average time interval between index fracture and the start of treatment with Teriparatide was 8.9 (range 2 – 27) months. When considering the time elapsed between the last procedure received, also intended as hardware breakage or failure as from a biomechanical point of view could be considered equivalent to an “auto-dynamization” of the fracture, and the start of therapy, a minimum time of 7 days and a maximum of 7 months was observed.

**Table 2:** Patients’ demographic and clinical details, treatment modalities and outcomes

N^d^	Gender	Age (years)	Fracture site	Type of pathology (delayed/nonunion)	Smoking	Alcohol	Diabetes	Previous fractures	Osteoporosis	Previous treatment	Administered therapy^d^	Duration of treatment (months)	Healing time (months)
1	F	78	Femur	Nonunion	No	Yes	No	Yes	Yes	Yes^b^	1	6	6
2	F	52	Femur	Delayed union	Yes	No	Yes	Yes	Yes	Yes^b^	1	6	6
3	M	18	Tibia	Delayed union	No	No	No	Yes	No	No	1	6	6
4	M	25	Femur	Delayed union	Yes	No	No	Yes	No^a^	No	1	6	6
5	F	70	Humerus	Delayed union	No	No	No	Yes	No	No	0	24	24
6	M	49	Forearm	Delayed union	Yes	No	No	Yes	No	No	0	6	6
7	M	79	Tibia	Delayed union	No	No	No	No	No	No	1	6	Lost to FU
8	M	26	Forearm	Delayed union	No	No	No	No	No	No	1	5	5
9	F	80	Femur	Delayed union	No	No	No	No	Yes	Yes^c^	1	4	4
10	M	37	Tibia	Delayed union	No	No	No	No	No	No	1	3	3
11	M	67	Femur	Delayed union	No	No	No	No	No	Yes^c^	1	5	5
12	F	82	Femur	Delayed union	No	No	No	Yes	Yes	Yes^b^	1	5	5
13	M	48	Femur	Delayed union	Yes	No	No	No	No	No	1	5	5
14	F	77	Femur	Delayed union	No	No	No	No	Yes	Yes^b^	1	19	6
15	F	72	Femur	Delayed union	Yes	No	Yes	Yes	Yes	No	1	5	7
16	M	35	Forearm	Delayed union	No	Yes	No	No	No	No	1	4	4
17	M	54	Femur	Delayed union	Yes	no	No	Yes	No	No	1	3	3
18	M	71	Femur	Delayed union	No	No	No	No	No	No	1	3	6
19	M	52	Femur	Delayed union	Yes	No	No	Yes	No	No	1	4	6
20	F	33	Femur	Delayed union	No	No	No	No	No	Yes^c^	1	6	6

*M* Male, *F* Female, *FU* Follow-up, *BPs* Bisphosphonates

^a^Mineralometric values indicated osteopenia

^b^Patient had received both BPs and vitamin D supplementation

^c^Patient had received vitamin D + calcium supplementation

^d^0 = Teriparatide 20 μg/die; 1 = Teriperatide 20 μg/die + Vitamin D and Calcium supplement according to vitamin D and calcium levels

**Table 3:** Differences between subgroups showing statistical differences in mean age of patients regarding to gender (male younger than females) and to osteoporosis (patients affected by osteoporosis older than non-affected ones)

a. Mean age (years)	55.3	±	20.9	(range 18 – 82)			
Gender	M			F			*p*
	46.8	±	19.3	68.0	±	17.0	**0.02521**
Comorbidities							
Osteoporosis	Yes			No			
	73.5	±	11.1	47.4	±	19.2	**0.006473**
Smoking	Yes			No			
	50.3	±	13.8	57.9	±	23.9	0.3832
Alcohol abuse	Yes			No			
	56.5	±	30.4	55.1	±	20.8	0.9498
Diabetes	Yes			No			
	62.0	±	14.1	54.5	±	21.7	0.7054
Previous fracture(s)	Yes			No			
	55.2	±	21.3	55.3	±	21.6	0.9698
b. Gender	M	12	(60%)	F	8	(40%)	
Comorbidities							
Osteoporosis	0		(0%)	6		(75%)	**0.0007**
Smoking	5		(41.7%)	2		(25%)	0.6424
Alcohol abuse	1		(8.3%)	1		(12.5%)	1
Diabetes	0		(0%)	2		(25%)	0.147
Previous fracture(s)	5		(41.7%)	5		(62.5%)	0.6499

*M* Male, *F* Female, in **bold** statistically significant results

At the time of the index fracture, 4 patients had already been treated with BPs which were discontinued before starting the treatment with Teriparatide. Hypovitaminosis D (25-hydroxyvitamin D less than 20 ng/mL) was found in 6 out of 20 patients (30%) before the start of therapy with Teriparatide. All patients were corrected by calcifediol or cholecalciferol oral supplementation [34–36]. Serum calcium and phosphorus levels did not change significantly, nor did blood creatinine levels during the entire treatment period. Mineralometric evaluation showed values ​​compatible with osteoporosis in 6 patients (30%) and with osteopenia in one patient (5%), and normal values in 13 patients (65%). The duration of therapy with Teriparatide ranged from 3 to 24 months, with a mean period of 6.6 ± 5.3 months. The median time to healing was 5.2 ± 1.1 months when considering patients healed within 6 months of starting therapy, and 6.3 ± 4.4 when considering all the patients recovered. Taking into consideration the patients’ gender, a non-statistically difference in the median time to healing was highlighted: 5.0 ± 1.2 months for men and 8.0 ± 6.5 months for women.

After 1 month of therapy, radiographic signs of favorable evolution were observed in 3 cases (15%), while no cases showed complete bone healing. At 3 months of therapy, complete fracture healing was observed in 2 patients (10%), partial improvement in 16 patients (80%), and no or poor improvement in 2 patients (10%). At 6 months, plain radiographs showed partial improvement in 3 patients (15%) and complete healing in 17 patients (85%) (*p* < 0.0001). Differences in bone healing were found with regard to gender of patients, although not statistically significant: 7 out of 8 females (87.5%) showed an improvement in the healing process but no case of complete healing at 3 months of therapy, while among males 2 out of 12 (16.7%) radiologically healed after 3 months of therapy (*p* = n.s.). At 6 months of therapy, 11 males (91.7%) completely healed while only 6 females (75%) did (*p* = n.s.). Also, some differences in the healing process were observed when considering comorbidities, but they were not statistically significant. Between osteoporotic patients (all females), none underwent complete recovery in the first three months of therapy but 5 out of 6 (83.3%) had a partial improvement (*p* = n.s.), while at 6 months 5 patients (83.3%) fully healed (*p* = n.s.). Out of the 10 patients with a history of previous fracture(s), 8 (80%) experienced a partial improvement and one (10%) achieved radiographic healing after 3 months of therapy (*p* = n.s.). At 6 months, 8 patients (80%) had healed (*p* = n.s.). All the smokers (7 patients) showed a partial improvement in the first 3 months of therapy (one of them was deemed radiographically healed) (*p* = n.s.), while at 6 months only one smoker (14.3%) had not yet completely healed (*p* = n.s.). Only one out of the 2 (50%) patients with a history of alcohol abuse showed an improvement in the pathological condition after the first 3 months of therapy (*p* = n.s.), but both healed completely after 6 months of therapy (*p* = n.s.).

**Fig. 1:**
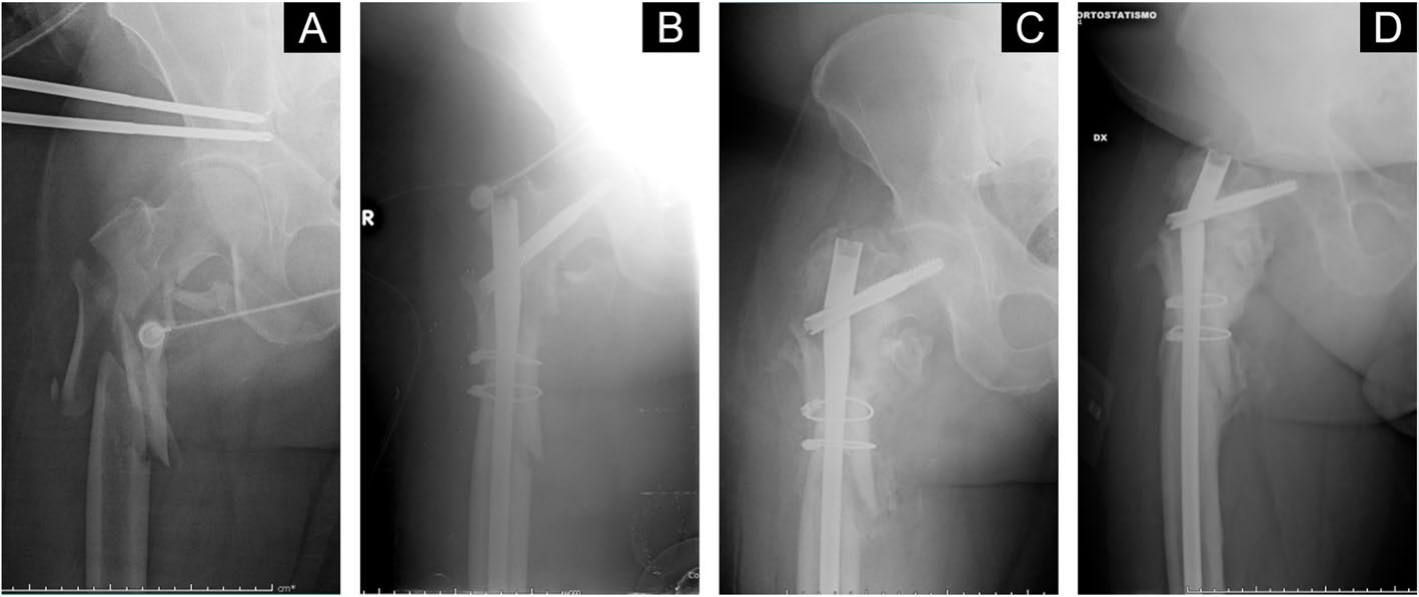
A case of a 45 years-old female who suffered from proximal femur fracture in a polytrauma scenario treated with urgent damage control (**A**) and subsequent cephalomedullary nailing (**B**), who experienced hardware breakage (**C**) at 4 months from surgery.  Teriparatide was administered and bone healing was evident at 6 months (**D**)

**Fig. 2:**
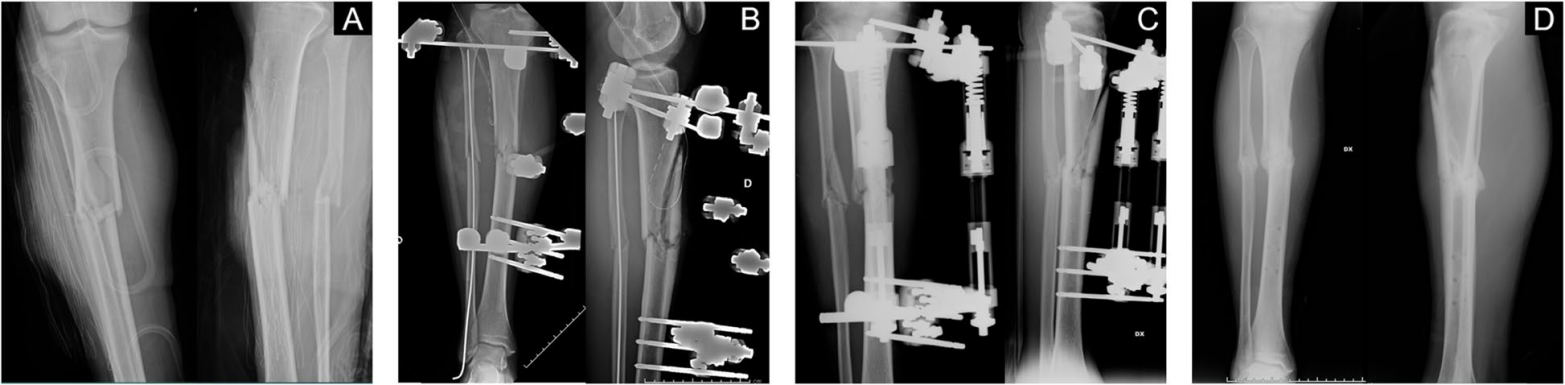
A case of a 50 years-old male who suffered from an open fracture of the right leg (**A**), treated with external fixation (**B**). Dynamization of the external fixator at 2 months from surgery and start of Teriparatide (**C**). Radiographic assessment after 3 months of therapy showed complete healing (**D**)

**Fig. 3:**
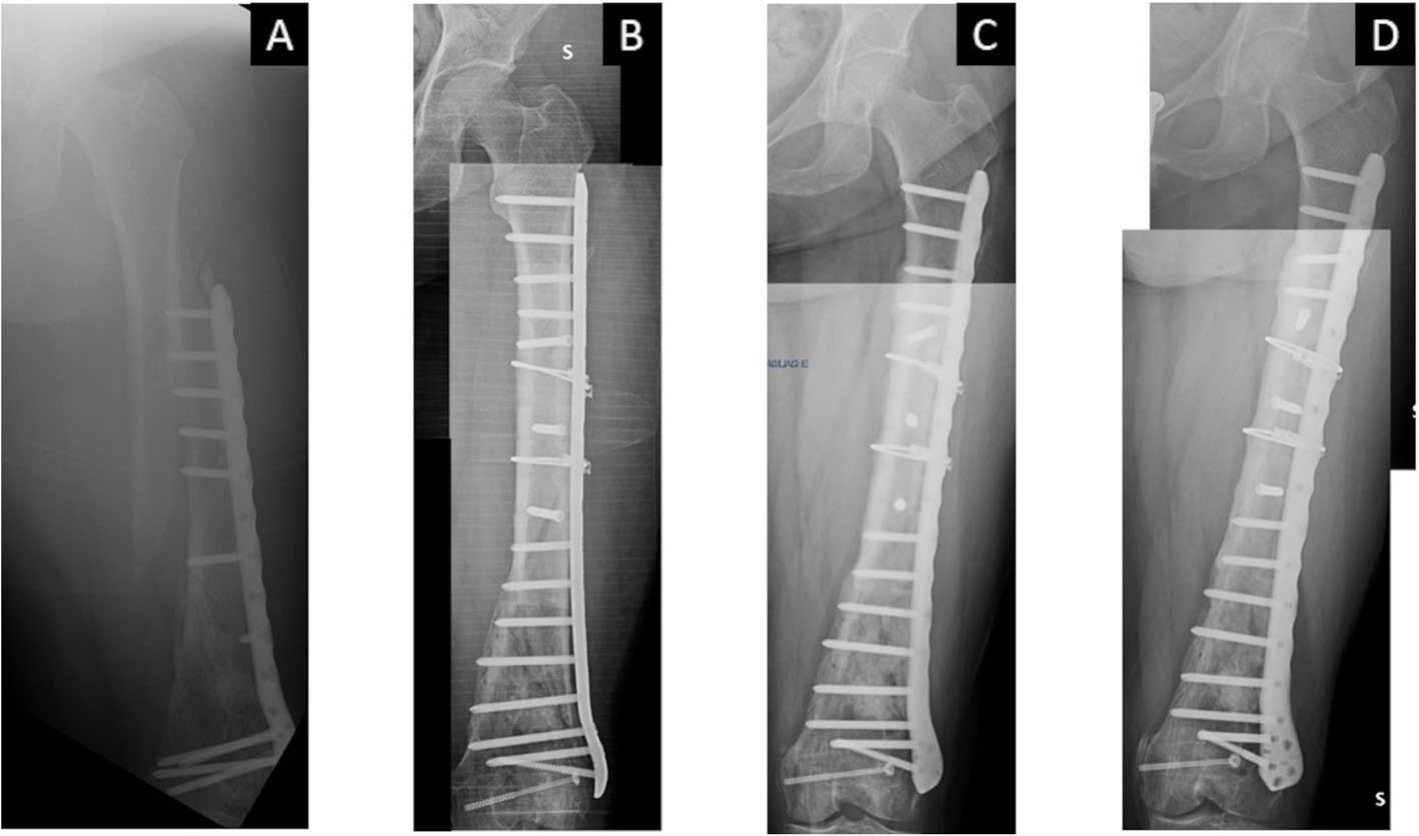
A case of a 51 years-old female who suffered from a peri-implant fracture of the left femur (**A**), who underwent revision surgery (**B**). Delayed union at 3 months from surgery (**C**) and start of Teriparatide. Complete healing at 3 months therapy (**D**)

Lastly, with regards to patients’ age, the mean age of the subjects who showed partial improvement in the first 3 months of therapy was 54.1 ± 21.8 years, while in the subjects who did not improve the mean age was 74.5 ± 4.9 years, and in those who were deemed radiographically healed the mean age was 45.5 ± 12.0 years (*p* = n.s.). At 6 months, patients who recovered had an average age of 52.0 ± 20.9 years, while those who did not undergo radiographic recovery had an average age of 73.7 ± 4.7 years (*p* = n.s.). In all patients, therapy was well tolerated, and no treatment interruptions were necessary due to the occurrence of side effects. Results are recapped in Tables 3, and 4 cases are shown in Figs. 1, 2 and  3.

**Table 4:** Radiographic results of the healing process at 1, 3 and 6 months after the start of therapy with Teriparatide^a^ in the study group

			1-month FU				3-months FU				6-months FU			
			NI	PI	CH	*p*	NI	PI	CH	*p*	NI	PI	CH	*p*
Gender		M	91.7	8.3	0	0.5368	8.3	75	16.7	0.746	0	8.3	91.7	0.5368
		F	75	25	0		12.5	87.5	0		0	25	75	
Comorbidities	Osteoporosis	Yes	66.7	33.3	0	0.202	16.7	83.3	0	1	0	16.7	83.3	1
		No	92.9	7.1	0		7.1	78.6	14.3		0	14.3	85.7	
	Smoking	Yes	71.4	28.6	0	0.2702	0	85.7	14.3	0.77	0	14.3	85.7	1
		No	92.3	7.7	0		15.4	76.9	7.7		0	15.4	84.6	
	Alcohol abuse	Yes	100	0	0	1	50	50	0	0.37	0	0	100	1
		No	83.3	16.7	0		5.6	83.3	11.1		0	16.7	83.3	
	Diabetes	Yes	50	50	0	0.28	0	100	0	1	0	50	50	0.28
		No	88.9	11.1	0		11.1	77.8	11.1		0	11.1	88.9	
	Previous fracture(s)	Yes	70	30	0	0.21	10	80	10	1	0	20	80	1
		No	100	0	0		10	80	10		0	10	90	

*NI* “No improvement”, *PI* “Partial improvement”, and * CH* “Complete healing” were defined according to the mRUST score. Differences in gender (male/female), and comorbidities (osteoporosis, history of previous fractures, smoking and alcohol abuse) were assessed, *M* Male, *F* Female, *FU* Follow-up

^a^All values are expressed as a percentage

Of the 3 patients who did not completely heal at 6 months, one patient with an oligosymptomatic nonunion of the tibia over an intramedullary nail was lost to follow-up at 1-year, a patient with an oligosymptomatic nonunion of the humerus prosecuted therapy for 24 months, with follow-up visits at 6-months interval, and eventually the fracture radiographically healed at last radiographical control. The last patient required re-intervention for a symptomatic nonunion of the distal femur with malalignment.

## Discussion

In general, about 5–10% of all fractures fail to heal, developing a nonunion whose symptomatology depends on various aspects, including patient’s level of activity and deambulatory status, other than the specific pattern of nonunion. A larger number of cases present delayed consolidation [34]. Both complications are the macroscopic expression of a lack of biological efficiency in bone healing process, the presence of a mechanical impairment, or both. They represent a major challenge for the orthopedic surgeon, also demonstrating a negative impact on health economics [37, 38]. The bone healing mechanism is triggered by a cascade of multiple factors that interact with each other [39]. Some of these are patient-dependent, such as age, osteoporosis, menopause, smoking, alcohol abuse and the use of drug such as BPs or GCs, but others depend on the trauma in its complexity and on the stability of fixation obtained [33].

Over time, many osteoporotic medications have been studied in bone healing, in delayed unions and nonunions [40]. Nowadays is largely validated, both in animal studies and in vitro, the use of Teriparatide as an anabolic drug effective in improving bone performance and preventing fractures in the osteoporotic patient. In accordance with recent Literature, it can also provide an extra weapon to the orthopedic surgeon who deals with fractures at risk of non-consolidation or nonunions, for the above-mentioned reasons [41–43].

The present study confirms the positive effect of Teriparatide in improving bone healing. In our previous experience [11, 15, 37], Teriparatide showed its maximum efficacy if associated with a stimulus that induces the revitalization of the healing process, such as re-intervention to refresh the nonunion site, dynamization in elastic construct (intramedullary nailing or external fixator), or even the failure with rupture of internal hardware that biomechanically represents a “self-dynamization” of the fracture [44]. Also, the use of pulse electromagnetic fields had shown a positive effect in improving bone healing in deleyed unions and nonunions [45]. However, Teriparatide seemed to be effective even in cases in which bone did not received any local mechanical or biological stimulus, if it is started early after surgery. In all cases in which we had early recovery at 3 months, Teriparatide had in practice been started immediately 7 days after the stimulus.

Current Literature suggests that there is no reason to believe that Teriparatide could only be effective in specific bones. However, in accordance with Literature where most case-reports concern the use of Teriparatide in long bones, we also used the drug in these types of fractures. This probably reflects the fact that delayed and nonunion is more common in long bones [46].

Compared with the use of Teriparatide in osteoporosis, where it is administered for a maximum duration of 2 years to maximize the anabolic effect of the drug, the use in bone healing after a fracture has been suggested for shorter periods. At 3 months of therapy, 80% of our patients showed a radiographic improvement, while 85% of patients had radiographically healed at 6 months. There was no need to prosecute the therapy in healed patients, except than in those with concomitant osteoporosis. In Literature, although the ideal duration of treatment depends on individual patients’ factors and the personality of the fracture, the duration of therapy is typically described to last between 2 and 9 months on average [17]. Regarding the maximum duration of treatment, however, since the use of Teriparatide for osteoporosis is only approved for a total duration of 24 months, it seems reasonable to apply this limit even in the off-label uses of this drug.

Several metabolic abnormalities have been associated with impaired fracture healing; most of them directly affect bone metabolism during the healing process and may have a prevalence of up to 50% in certain populations [47]. For this reason, a study of the phosphocalcic metabolism prior to the prescription of Teriparatide is mandatory, to identify and correct any eventually present abnormality such as hypovitaminosis D, other than to exclude potential contraindications to its administration. All contraindications reported in the drug data sheet were observed. Moreover, because of the proposed use was an off-label indication, we broadened exclusion criteria (infections and previous oncologic diseases not affecting bone) for higher safety.

As for the union rate, in the present study bone healing occurred in 85% at 6 months and in 95% at 24 months, comparing to existing literature that suggest union rate as high as 95% [36]. Also, in the present study it appears that bone healing was achieved in shorter time in male patients; however, it must be taken into consideration that mean age among females was higher that among males. Moreover, among elderly abnormal bone metabolism and compromised bone healing are often present and can be considered predisposing factors of delayed healing [48, 49]. Despite the small sample size, no significant difference between patients affected or not by prior fractures was observed; no difference emerged between smokers and nonsmokers either, nor in relation to alcohol abuse. Lastly, no adverse events were reported, in accordance with the review of Canintika who concluded that Teriparatide application in nonunions and delayed unions is safe [38].

Limitations are present in the current study. It is a retrospective study with a limited number of patients with miscellaneous district of nonunions and non-homogeneous characteristic. It may be difficult to correctly generalize the data obtained because of the small sample size. Moreover, the low patients’ number limits the power of the statistical analysis performed, and as a result, differences that could otherwise be identified in a larger sample have not necessarily been detected.

## Conclusion

When a fracture shows signs of difficulty in healing in time (delayed union) or in cases of overt nonunion, all available surgical and non-surgical treatment strategies need to be employed. Among the latter, systemic anabolic support with Teriparatide has already proved its effectiveness, and the present study confirms the positivity of this trend in the Literature. From our experience, a greater effect of the drug emerges when associated with a condition in which the bone is in an “active” phase of callogenesis, linked to a revitalizing treatment such as a re-intervention or dynamization, which represents a local mechanical and biological stimulus to the healing process.

Despite the low number of patients involved, this study showed a significant improvement in the pathological condition of subjects suffering from nonunion during the months of treatment with Teriparatide, highlighting how this therapy can play a leading role in the treatment of this pathology. Although the results obtained are encouraging, further studies, particularly prospective and randomized, are necessary to confirm the efficacy of the drug and define a specific treatment algorithm.

## Data Availability

The datasets used and/or analyzed during the current study are available from the corresponding author on reasonable request.
